# A Need for Honoring Healthcare Retirees: Proposed Recommendations

**DOI:** 10.34172/ijhpm.8455

**Published:** 2024-10-07

**Authors:** Fatemeh Shaygani, Behnam Honarvar, Farahnaz Izadi, Fatemeh Rafiee

**Affiliations:** ^1^Student Research Committee, Shiraz University of Medical Sciences, Shiraz, Iran.; ^2^Health Policy Research Center, Institute of Health, Shiraz University of Medical Sciences, Shiraz, Iran.

## Dear Editor,

 The retirement of healthcare professionals marks a significant milestone after years dedicated to the care and well-being of others. Their tireless efforts and invaluable contributions have left an indelible mark on the healthcare landscape.^1,2^ Additionally, their positive influence extended beyond patients and colleagues to policy-makers and the general population, underscoring the importance of recognizing and appreciating their impact.^3,4^

 As the healthcare industry evolves, it is increasingly important to acknowledge and honor the accomplishments and expertise of retired healthcare professionals.^4-6^ Globally, various strategies have been implemented to recognize and support healthcare retirees, including recognition ceremonies, retirement celebrations, personalized gifts, and pension loans.^7,8^ However, these initiatives often require substantial budgets, posing challenges for countries with limited financial resources, such as low- and middle-income countries. Therefore, we recommend alternative strategies that can be implemented with more modest budgets. Highlighting retirees’ accomplishments and enabling their continued involvement are crucial strategies for health policy-makers, particularly in developingcountries with restricted healthcare budgets.

 One effective approach to honoring retirees is to highlight their accomplishments by sharing invaluable experiences which they gained over the decades of dedicated service. These experiences can provide valuable insights and guidance to younger individuals embarking on their careers in the same fields. To facilitate this, retirees’ experiences can be recorded, classified and documented into video, audio, or written files. The retirees’ preference for how they hand their experience and knowledge (via video, audio, or written documents) is also important. Additionally, periodic online or in person meetings can be arranged to tap into the expertise of retired individuals for the benefit of new employees. These files can be shared with current department heads, ensuring that the recorded experiences are easily accessible to younger individuals and others in related roles. Implementing this strategy creates a valuable information package which can enhance the quality of services which are provided by new healthcare workers. Additionally, it preserves the dignity of retirees, demonstrating that their experiences are highly regarded, considered valuable and will be used even after their retirement. This project incurs minimal costs for organizations, and can provide a valuable archive over time, documenting the retirees’ experiences comprehensively and enduringly.

 Another important strategy is to encourage the ongoing involvement of healthcare retirees within society. This can be achieved by creating opportunities for retired professionals to remain connected and engaged through mentorship programs, guest speaking engagements, or volunteering within healthcare organizations. Retirees’ participation in the health policy-making think tanks can also be highly beneficial, as their experience, wisdom, and diverse perspectives can enhance decision-making for comprehensive and effective healthcare strategies. By leveraging their expertise and experience, retirees can contribute to the betterment of the community, reduce costs and make a positive impact in the field of healthcare.

## Conclusion

 Retired healthcare professionals often feel that they have been overlooked despite their past valuable contributions. Properly honoring retirees involves recognizing their ongoing worth and establishing their legacy in the healthcare system. This recognition ensures that their expertise continues to benefit others. This letter provides recommendations for enhancing retirees’ experiences and highlights the benefits of their contributions to patients, colleagues, students, and healthcare managers. Additionally, it suggests conducting practical research to gain insights and address these issues effectively.

## Ethical issues

 Not applicable.

## Conflicts of interest

 Authors declare that they have no conflicts of interest.

## References

[1] Mäcken J, Merkel S, Heß M, Hilbert J, Naegele G (2019). Transition to retirement in the healthcare sector: working conditions and attitudes of older workers. Z GerontolGeriatr.

[2] Perera S, Martínez M, Monreal-Bosch P (2013). Managing the process of retirement: the medical professionals’ perceptions. Procedia Soc Behav Sci.

[3] Crego A, Alcover de la Hera C, Martínez-Íñigo D (2008). The transition process to post-working life and its psychosocial outcomes. Career Dev Int.

[4] Stattin M, Bengs C (2022). Leaving early or staying on? Retirement preferences and motives among older health-care professionals. Ageing Soc.

[5] Hewko S, Reay T, Estabrooks CA, Cummings GG (2019). The early retiree divests the health workforce: a quantitative analysis of early retirement among Canadian Registered Nurses and allied health professionals. Hum Resour Health.

[6] Helal DH, do Nóbrega CV, de Lima TA (2021). Retirement and organizations: perspectives and challenges for both workers and human resource management. Work Older People.

[7] Wilson LB, Lester L, Simson SP (2000). Assessing the involvement of retired health professionals in meeting the needs of underserved populations. Nurs Outlook.

[8] Alcover CM, Topa G, Fernández JJ (2014). Organizational management of older workers and the processes of maintaining, extending and leaving employment. Pap Psicol.

